# Degradation of Tetracycline with BiFeO_3_ Prepared by a Simple Hydrothermal Method

**DOI:** 10.3390/ma8095310

**Published:** 2015-09-18

**Authors:** Zhehua Xue, Ting Wang, Bingdi Chen, Tyler Malkoske, Shuili Yu, Yulin Tang

**Affiliations:** 1 State Key Laboratory of Pollution Control and Resource Reuse, College of Environmental Science & Engineering, Tongji University, Shanghai 200092, China; E-Mails: xxzzhh1990@126.com (Z.X.); wangting@163.com (T.W.); tylermalkoske@gmail.com (T.M.); ysl@tongji.edu.cn (S.Y.); 2 The Institute for Advanced Materials and Nano Biomedicine, Tongji University, Shanghai 200092, China; E-Mail: inanochen@tongji.edu.cn

**Keywords:** tetracycline, bismuth ferrite, visible light photocatalysis, Fenton, photo-Fenton

## Abstract

BiFeO_3_ particles (BFO) were prepared by a simple hydrothermal method and characterized. BFO was pure, with a wide particle size distribution, and was visible light responsive. Tetracycline was chosen as the model pollutant in this study. The pH value was an important factor influencing the degradation efficiency. The total organic carbon (TOC) measurement was emphasized as a potential standard to evaluate the visible light photocatalytic degradation efficiency. The photo-Fenton process showed much better degradation efficiency and a wider pH adaptive range than photocatalysis or the Fenton process solely. The optimal residual TOC concentrations of the photocatalysis, Fenton and photo-Fenton processes were 81%, 65% and 21%, while the rate constants of the three processes under the same condition where the best residual TOC was acquired were 9.7 × 10^−3^, 3.2 × 10^−2^ and 1.5 × 10^−1^ min^−1^, respectively. BFO was demonstrated to have excellent stability and reusability. A comparison among different reported advanced oxidation processes removing tetracycline (TC) was also made. Our findings showed that the photo-Fenton process had good potential for antibiotic-containing waste water treatment. It provides a new method to deal with antibiotic pollution.

## 1. Introduction

Tetracycline (TC) as a representative antibiotic is extensively used in human and veterinary medicine and is toxic to aquatic organisms [1,2,3]. An estrogenic effect of TC has also been discovered [4,5]. TC residues may promote the development of antibiotic-resistant microorganisms [6,7]. The existence of TC in natural water bodies may pose serious threats to the ecosystem and human health. Therefore, the removal of TC from the environment has become an important issue.

As far as we know, the conventional physical and chemical water treatment processes lack adequate removal efficiency of TC [8,9]. Fortunately, photocatalytic processes provide a good way for TC degradation. In the past few years, some research on the highly-efficient photocatalytic degradation of TC by different photocatalysts was reported [10,11]. However, most of the photocatalysts are UV-light driven, rather than visible light driven. Therefore, new types of visible light-driven photocatalysts with high efficiency for TC degradation are still desirable [12]. In addition, heterogeneous Fenton oxidation has also been used to remove TC [13,14].

BiFeO_3_ (BFO) has been regarded as one of the promising visible light photocatalysts for the degradation of organic pollutants [15,16,17,18]. It can also act as a heterogeneous Fenton catalyst [19,20]. Recently, research groups have developed various methods to prepare BFO as a catalyst to remove dye, pesticide, and so on [15,17,21,22]. To the best of our knowledge, no reports of visible light-driven photocatalysis and Fenton degradation of TC or other antibiotics by BFO have been published.

The objective of the present work is to evaluate the reaction activity of TC degradation under photocatalysis, Fenton and photo-Fenton processes catalyzed by BFO. The influences of various operation parameters, such as BFO concentration, initial pH and H_2_O_2_ concentration, on the reaction were investigated, and the mechanism was also discussed. This research not only optimizes the degradation process of TC, but also provides a new method to deal with antibiotic pollution.

## 2. Results and Discussion

### 2.1. Materials Characterization

**Figure 1:**
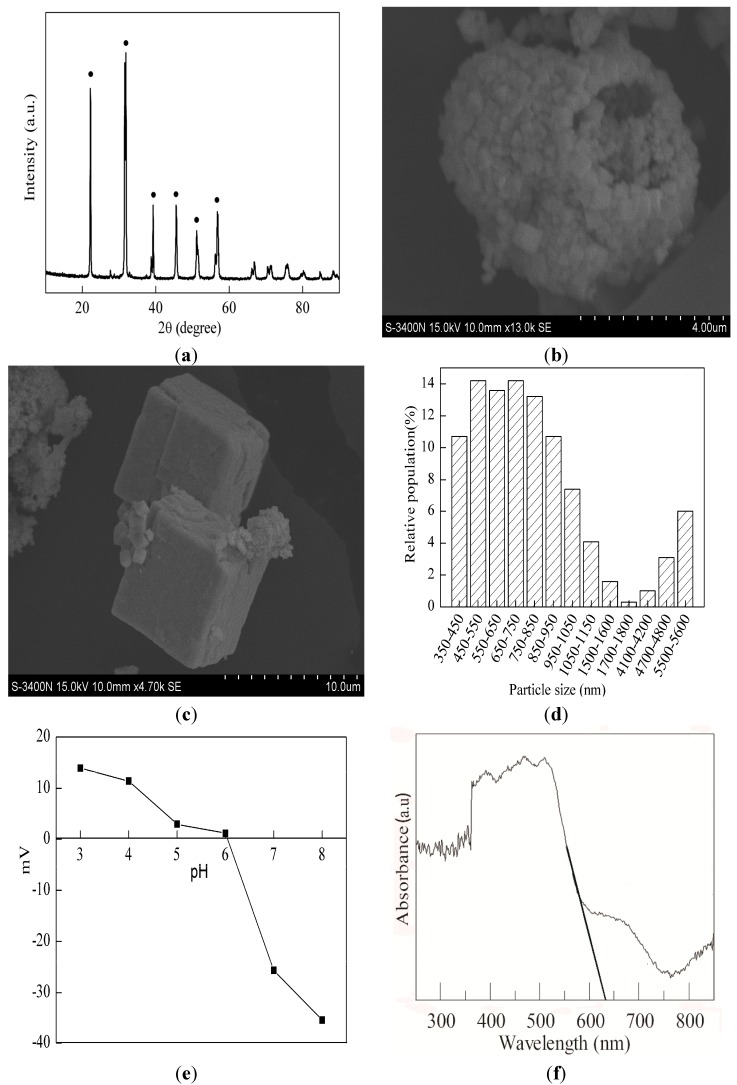
(**a**) XRD pattern of BiFeO_3_ (BFO); (**b**,**c**) SEM images of BFO; (**d**) Particle size distribution of BFO; (**e**) Zeta potential of BFO at different pH values; (**f**) UV-VIS spectra of BFO.

The XRD pattern of the BFO photocatalyst is shown in Figure 1a. The diffraction peaks were identified at 22.2°, 31.8°, 39.3°, 45.6°, 51.1° and 56.8°, which are assigned to the perovskite phase of bismuth ferrite (JCPDS 86-1518). According to the XRD results, BFO was successfully prepared.

BFO had a relatively smooth morphology as a regularly-cubic crystallite from the SEM image in Figure 1b,c. The size distribution and zeta potential of the BFO are shown in Figure 1d,e. The isoelectric point of BFO catalyst is nearly 6.0. BFO has a wide size distribution range from 342 to 5560 nm.

The UV-VIS adsorption spectrum of BFO is shown in Figure 1f. It can be seen that the adsorption edge (λ_g_) of BFO is in the visible light region (λ > 400 nm). BFO has visible light-responding properties. Drawing a tangent line on the adsorption curve where adsorbance has an abrupt drop and extending the line to intersect the horizontal axis, it can be roughly judged from the point of intersection that the adsorption edge of prepared BFO is 630 nm. Therefore, the energy gap of BFO is estimated to be 1.97 eV [23].

### 2.2. Adsorption Test

An adsorption test was performed to determine the adsorption equilibrium time of BFO. The structural moieties and pH-dependent speciation of TC with different pKa values was reported [24]. In the pH range of 3.0 to 8.0, TC was dominantly present in a form from positive ions, neutral molecules to negative ions. At the same time, the surface of BFO catalyst is positively and negatively charged, respectively, at pH = 3.0 and 8.0. There will be repulsive force between BFO and TC at pH = 3.0, which cause it to take longer for TC to reach adsorption equilibrium. Therefore, the initial pH values for the adsorption test were selected to be 3.0.

**Figure 2:**
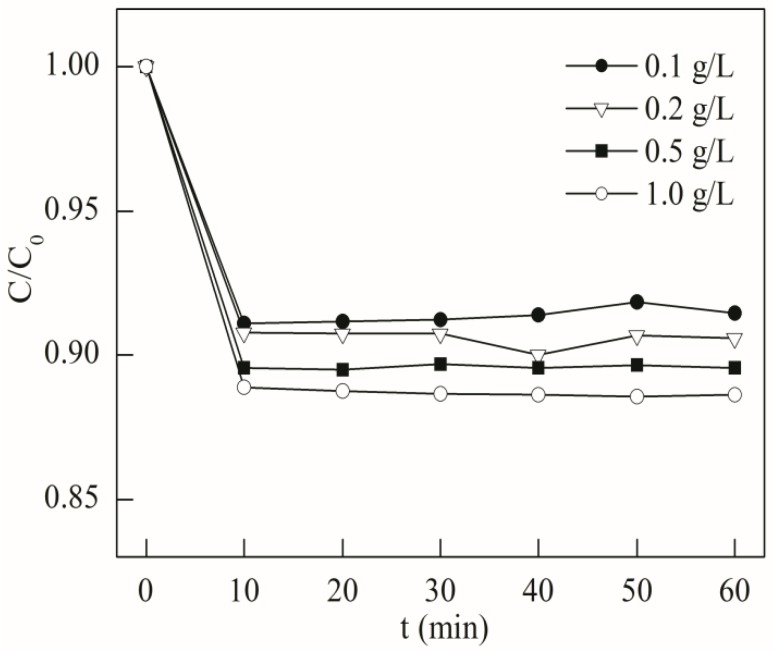
Tetracycline (TC) adsorption by BFO with different dosages (initial TC concentration = 10.0 mg/L, pH = 3.0).

The sampling interval is 10 min with a duration of 60 min in this test, and the results are shown in Figure 2. In the first 10 min, the residual TC concentration dropped to about 90% with different initial BFO concentrations, and little change in residual concentration can be seen. Though there was a slight drop in residual concentration as the BFO dosage increased, this change was not significant. With BFO dosage increasing from 0.1 to 1.0 g/L, the residual concentration only dropped from about 91% to 88%. Therefore, TC adsorption equilibrium can be achieved within 60 min.

### 2.3. Photodegradation of Tetracycline

#### 2.3.1. Effect of BFO Dosage

**Figure 3:**
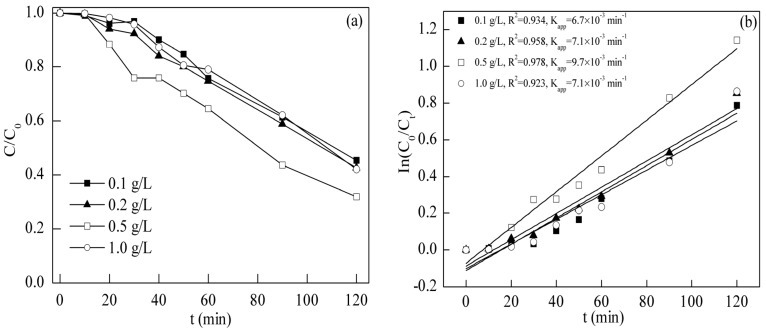
(**a**) Degradation and (**b**) removal kinetics of TC at different BFO dosages under visible light irradiation (initial TC concentration = 10.0 mg/L, pH = 3.0, time = 120 min).

The photocatalytic activities of BFO were evaluated by the degradation of TC under visible light irradiation. The effect of BFO dosage within a range from 0.1 to 1.0 g/L was investigated. As shown in Figure 3, when the BFO concentrations were 0.1, 0.2 and 0.5 g/L, the final residual TC concentrations were 45%, 42% and 31%. This revealed that the photo degradation efficiency increased within the concentration range from 0.1 to 0.5 g/L. However, as the BFO concentration kept going up to 1.0 g/L, the residual concentration was 42%, implying a decrease in photocatalytic efficiency with an increase in BFO concentration. To further quantify and express the change of TC removal with the variation of the BFO dosage, pseudo-first order kinetics was used to fit the photocatalytic results under different BFO dosages. This kinetics can be expressed as ln(*c*_t_/*c*_0_) = *k*_app_*t* + *y*, where *y* is a constant, *t* is the reaction time (min), *k*_app_ is the apparent rate constant (min^−1^) and *c*_0_ and *c*_t_ are the TC concentrations (mg/L) at time of *t* = 0 and *t* = *t*. The apparent rate constants (*k*_app_), shown in Figure 3, were 6.7 × 10^−3^, 7.1 × 10^−3^, 9.7 × 10^−3^, 7.1 × 10^−3^ min^−1^ for BFO dosages of 0.1, 0.2, 0.5, 1.0 g/L, respectively. All of the correlation coefficients *R*^2^ were higher than 0.9, indicating that the pseudo first order kinetics model fit the experimental data well. The results also revealed that the photocatalytic activity was the highest at a BFO dosage of 0.5 g/L. The decline in the degradation of TC may be due to the growth of turbidity with BFO concentration increasing, which inhibited light penetration [25]. Therefore, the optimal dosage for the following photocatalysis experiment was selected as 0.5 g/L.

#### 2.3.2. Effect of Initial pH

The reaction mechanism of the variation of photocatalytic efficiency with the change of pH has been studied [26,27]. The reaction formula can be summarized as follows:

(1) $$PC\overset{hv}{\to }h^{+}+e^{-}(PC:photocatalyst)$$

(2) $$H_{2}O+h^{+}\to \cdot OH+H^{+}$$

(3) $$OH^{-}+h^{+}\to \cdot OH$$

(4) $$O_{2}+e^{-}\to \cdot {O_{2}}^{-}$$

(5) $$\cdot {O_{2}}^{-}+H^{+}\to \cdot HO_{2}(pKa=4.88)$$

(6) $$\cdot HO_{2}+\cdot HO_{2}\to H_{2}O_{2}+O_{2}$$

(7) $$\cdot {O_{2}}^{-}+\cdot HO_{2}\to H{O_{2}}^{-}+O_{2}$$

(8) $$H{O_{2}}^{-}+H^{+}\to H_{2}O_{2}$$

**Figure 4:**
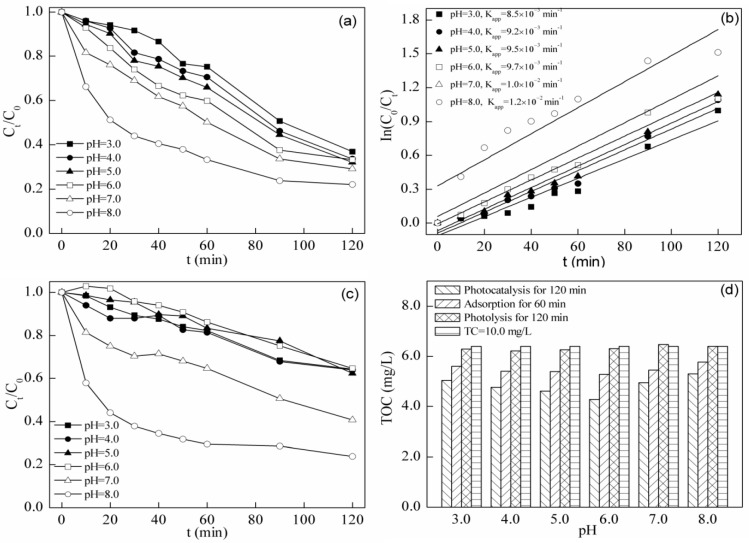
(**a**) Degradation and (**b**) removal kinetics of TC with BFO under visible light irradiation at different pH values; (**c**) Degradation of TC under visible light irradiation without BFO at different pH values; (**d**) Residual TOC after photolysis, adsorption and photolysis (initial TC concentration = 10.0 mg/L, initial BFO concentration = 0.5 g/L).

Equations (1)–(3) show that the ·OH radical formation under light excitation is caused by the positive holes reacting with H_2_O and OH^−^ on the photocatalyst surface [28,29]. If H^+^ ions are too high in concentration in the acidic condition, the excitation of H_2_O and OH^−^ into ·OH radicals will be suppressed due to an excessive concentration of H^+^ and a low concentration of OH^−^. Furthermore, the reaction in Equation (5) proceeds inversely and, thus, is inhibited when the pH exceeds the pK_a_. As a result, there will be less ·HO_2_ radicals, which are lower in redox potential and oxidizing capacity in the reaction system. Furthermore, the lack of ·HO_2_ radicals suppresses Equations (6)–(8), and these reactions will also inhibit oxidation, as they produce oxidizing substances lower in oxidizability. To summarize, the photocatalyst will show better oxidizing capacity at neutral pH or higher [30,31,32,33]. As can be seen in Figure 4a,b, the residual concentrations of TC for pH values ranging from 3.0 to 6.0 were all around 35% at 120 min, and the apparent rate constants were 8.5 × 10^−3^, 9.2 × 10^−3^, 9.5 × 10^−3^ and 9.7 × 10^−3^ min^−1^. This means that the photocatalysis efficiency remains unchanged within this pH range. However, when the pH value rose to 8.0, the final concentration of TC reduced to 22%, and the rate constant increased to 1.2 × 10^−2^ min^−1^. This phenomenon was seemingly in accordance with the theory mentioned that photocatalytic performance will be better at neutral pH or higher [26,27].

It was noteworthy that the correlation coefficient of degradation results when pH = 8.0 fit by pseudo first order kinetics was below 0.9, while that of the results at other pH values was above 0.9. It is rational to regard that some reaction other than photocatalysis makes the results inappropriate to be fitted by pseudo first order kinetics.

The results of a blank test conducted without photocatalyst are shown in Figure 4c. TC will be degraded solely by irradiation of visible light, and the effect was pH relevant. The residual concentration of TC after 120 min was around 60% in a pH range from 3.0 to 6.0, while that at pH = 8.0 was 23%, which reflected that visible light photolysis could cause the degradation of TC [34,35,36,37,38]. With the increase of pH, the adsorption spectrum of TC exhibits a red shift. Due to the shift of the adsorption spectrum to a visible light region with pH values rising, the number of photons adsorbed per unit time increased, which resulted in the higher photolysis efficiency at higher pH. The residual concentrations of the blank test were higher than those of the photocatalysis tests at pH values from 3.0 to 7.0. The initial concentrations for photocatalysis and the blank test were almost the same, because the adsorption test revealed that BFO had a low adsorption capacity. If other particles were added rather than the photocatalyst, the turbidity increase is supposed to weaken the photolysis. Therefore, this enhancement of degradation efficiency by adding BFO means that photocatalysis actually plays a part in the degradation. When the pH value was 8.0, the results of the two tests were almost the same. The degradation results above came from chromatography measurement. As can be seen from the results, the degradation efficiencies of both photolysis and photocatalysis on TC are pH dependent.

The actual effect of visible light photocatalysis degradation of TC under different pH conditions was hard to evaluate solely by using chromatography measurement as the standard. Therefore, TOC measurement was adopted in order to judge the change of photocatalysis efficiency. According to Figure 4d, TOC concentration values after photolysis between the results from the experimental groups with different initial pH values showed almost no change. After 60 min of adsorption, the residual TC concentration at pH = 6.0 was the lowest. The isoelectric point of BFO is 6.0. Therefore, the repulsive force between the TC molecule and BFO is the lowest at pH = 6.0, which makes the adsorption capacity the highest at pH = 6.0. When it comes to the residual TOC concentration of TC after being degraded by photocatalysis at 120 min, the best result was also at pH = 6.0. The phenomenon of optimal adsorption and photocatalysis removal at the same pH = 6.0 instead of at higher pH may be because TC adsorption on the photocatalyst decreases with the pH increasing, which makes ·OH radicals entering the solution the rate determining step, which decreased the TC degradation at higher pH [39]. The best degradation efficiency of TC was achieved at pH = 6.0 according to the TOC results. Many kinds of pollutants tend to be degraded by photolysis when measured by chromatography [17,40]. TOC measurement may be a good way to exclude the influence from visible light photolysis and to achieve the goal of evaluating the actual degradation efficiency of visible light photocatalysis, because it may be hard for visible light photolysis to bring about TOC reduction. The TOC measurement was adopted in all of the following degradation tests in this study.

### 2.4. Fenton Degradation of Tetracycline

#### 2.4.1. Effect of H_2_O_2_ Dosage

**Figure 5:**
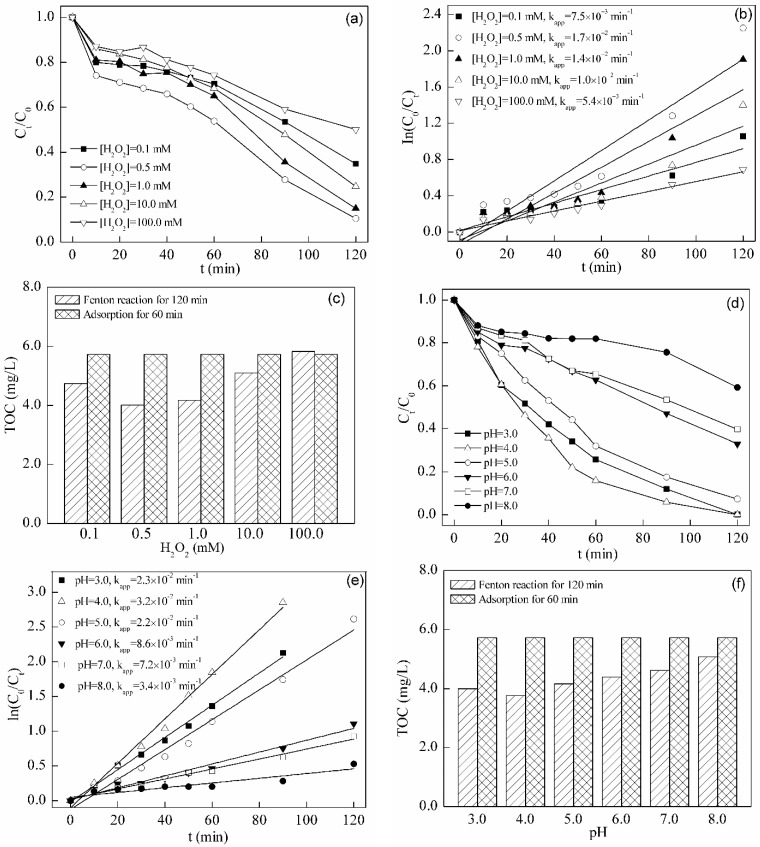

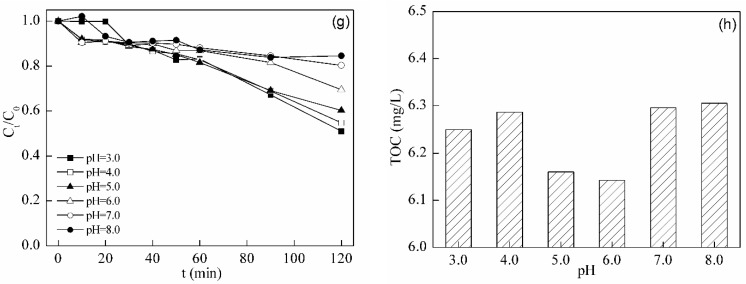
(**a**) Degradation and (**b**) removal kinetics of TC in the BFO-catalyzed Fenton-like system at different H_2_O_2_ dosages; (**c**) Residual TOC after adsorption and Fenton-like degradation (initial TC concentration = 10.0 mg/L, initial BFO concentration = 0.5 g/L, pH = 3.0); (**d**) Degradation and (**e**) removal kinetics of TC in the BFO-catalyzed Fenton-like system at different initial pH values; (**f**) Residual TOC after adsorption and Fenton-like degradation (initial TC concentration = 10.0 mg/L, initial BFO concentration = 0.5 g/L, initial H_2_O_2_ concentration = 0.5 mM); (**g**) Degradation and (**h**) residual TOC of TC with H_2_O_2_ only at different pH values in the dark (initial TC concentration = 10.0 mg/L, initial H_2_O_2_ concentration = 0.5 mM, time = 120 min).

The degradation capacity of the Fenton-like system catalyzed by BFO on TC was evaluated. The Fenton-like system used a BFO dosage of 0.5 g/L and a pH of 3.0 [19,20,41]. The initial TC concentration was 10.0 mg/L, and the H_2_O_2_ dosages were chosen to be 0.1, 0.5, 1.0, 10.0 and 100.0 mM. According to Figure 5, the best dosage of H_2_O_2_ was 0.5 mM. The apparent rate constant fitted by pseudo first order kinetics was the highest, and the residual concentration of TC was the lowest, as shown in Figure 5a,b. The residual TOC concentration after 120 min of Fenton-like degradation in Figure 5c was in accordance with the result from chromatography. Final TOC concentrations at a H_2_O_2_ dosage of 0.5 mM were the lowest. The mechanism of the heterogeneous Fenton-like system excited by iron-containing catalysts has been suggested to proceed via the following reactions [19,42,43,44,45]:

(9) $$Fe{\left(\mathrm{ΙΙΙ}\right)}_{surf}+H_{2}O_{2}\to Fe{\left(\mathrm{ΙΙΙ}\right)}_{surf}\;(H_{2}O_{2})$$

(10) $$Fe{\left(\mathrm{ΙΙΙ}\right)}_{surf}(H_{2}O_{2})\to Fe{\left(\mathrm{ΙΙ}\right)}_{surf}\;+\cdot HO_{2}+H^{+}$$

(11) $$\cdot HO_{2}↔\cdot {O_{2}}^{-}+H^{+}(pKa=4.8)$$

(12) $$Fe{\left(\mathrm{ΙΙΙ}\right)}_{surf}+\cdot HO_{2}/\cdot {O_{2}}^{-}\to Fe{\left(\mathrm{ΙΙ}\right)}_{surf}+O_{2}(+H^{+}\text{ )}$$

(13) $$Fe{\left(\mathrm{ΙΙ}\right)}_{surf}+H_{2}O_{2}\to Fe{\left(\mathrm{ΙΙΙ}\right)}_{surf}+\cdot OH+OH^{-}$$

(14) $$\cdot OH+H_{2}O_{2}\to \cdot HO_{2}+H_{2}O$$

(15) $$TC+\cdot OH\to \cdot \cdot \cdot CO_{2}+H_{2}O$$

First, H_2_O_2_ forms a complex with Fe(III) sites at the catalyst surface in Equation (9). Afterwards, Fe(III) sites in this complex are converted to Fe(II) sites in Equation (10). Surface Fe(II) reacts with H_2_O_2_ to form ·OH and Fe(III) in Equations (12) and (13). Excessive H_2_O_2_ will react with ·OH and produce ·HO_2_ with weaker oxidation capacity. This can explain the decrease of oxidation efficiency under the over-dose of H_2_O_2_ in this test. Figure 5g,h shows the degradation of TC by H_2_O_2_ only. The removal rate, reaction speed and TOC removal drop without BFO. BFO indeed functions as a catalyst in this test. The best dosage pair of BFO and H_2_O_2_ according to the test was 0.5 g/L and 0.5 mM, respectively. Compared to the results from the photocatalysis test, both the optimal reaction rate and TOC removal were better in the Fenton-like degradation system. Therefore, BFO was better as a kind of heterogeneous Fenton catalyst than a photocatalyst.

#### 2.4.2. Effect of Initial pH

One of the most important parameters that influence the Fenton degradation of TC is pH. Under the optimized dosage pair of BFO and H_2_O_2_, a study on the influence of pH on the degradation efficiency of BFO on the excited Fenton-like system was performed in a pH range from 3.0 to 8.0. As shown in Figure 5d,e, in a pH range from 3.0 to 5.0, the residual concentrations of TC at 120 min are below 10%, and the apparent rate constants are above 2.2 × 10^−2^ min^−1^. The results of residual TOC concentration also reveal a good performance within this pH range in Figure 5f. Although the efficiency of the classic homogeneous Fenton system is also high, it only operates at pH < 3.0 [42,46]. The heterogeneous Fenton-like system in this test can achieve good performance at pH values higher than 3.0. Therefore, it may save the expense of pH adjustment by substituting the BFO heterogeneous Fenton-like system for the classic homogeneous one. With increasing pH value, the degradation efficiency was weakened in Figure 5d,f. The apparent rate constant dropped, and residual TOC grew. During the experiment, more bubbles could be seen in the reaction solution at higher pH values, which were induced by higher O_2_ production from the self-decomposition of H_2_O_2_. Therefore, the decline in the degradation efficiency was likely due to the self-decomposition of H_2_O_2_ at the higher pH conditions. In addition, the higher oxidative capacity may be attributed to more ·OH production under the acidic condition [19].

### 2.5. Photo-Fenton Degradation of Tetracycline

**Figure 6:**
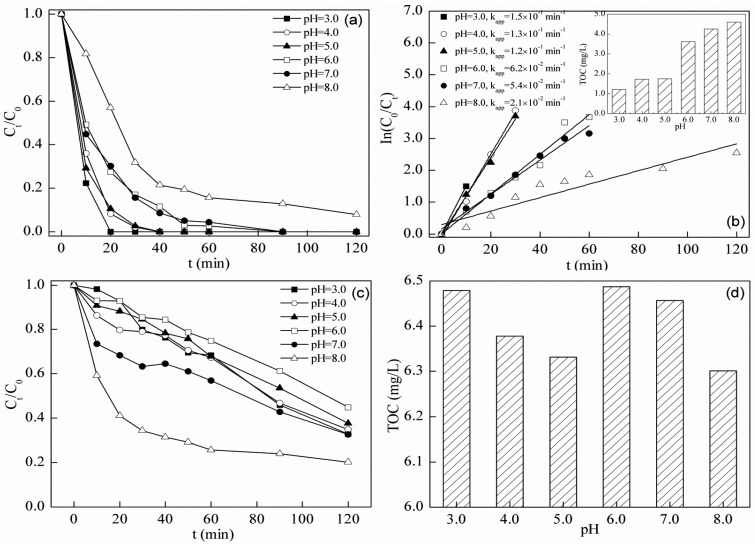
(**a**) Degradation; (**b**) removal kinetics and residual TOC (inset) of TC in the BFO-catalyzed photo-Fenton system at different pH values (initial TC concentration = 10.0 mg/L, initial BFO concentration = 0.5 g/L, initial H_2_O_2_ concentration = 0.5 mM); (**c**) Degradation and (**d**) residual TOC concentration of TC with H_2_O_2_ at different pH values (initial TC concentration = 10.0 mg/L, initial H_2_O_2_ concentration = 0.5 mM, time = 120 min).

Photocatalysis and Fenton-like degradation of TC by BFO were both effective to some extent. Moreover, the combination of these two processes will significantly enhance the degradation efficiency in Figure 6. The performance of the process was significantly enhanced in the photo-Fenton system compared with the results from photocatalysis and the Fenton-like system. The apparent rate constant and residual TOC results from photocatalysis, Fenton-like and photo-Fenton processes at different pH conditions are shown in Table 1. Blank tests in Figure 5g,h and Figure 6c,d show that, under conditions without BFO, the degrading effects could not reach the level achieved by the photo-Fenton test. The addition of BFO led to the formation of a catalytic system enhancing the effect. The optimal results of the photo-Fenton process were much better than those from the other two. The photo-Fenton process showed synergistic joint effects of photocatalysis and the Fenton process. The oxidation of TC in a photo-Fenton system is likely caused by several mechanisms [47]: (i) excitation of H_2_O by the electron-hole pair into radicals; (ii) excitation of H_2_O_2_ by electron-hole pairs into radicals; and (iii) excitation of H_2_O_2_ by surface Fe into radicals. The apparent rate constant and residual TOC concentration were 6.2 × 10^−2^ min^−1^ and 63%, even at pH = 6.0, in the photo-Fenton system. At lower pH values, the performance was even better. In the Fenton-like system, optimal *k*_app_ was 3.2 × 10^−2^ min^−1^, and the residual TOC concentration was 65% at pH = 4.0. The photo-Fenton process revealed stronger catalytic activity in a wider operational pH range. This system is promising for application in antibiotic-containing waste water treatment.

**Table 1:** The apparent rate constants (*k*_app_) and residual TOC concentration for photocatalysis, the Fenton process under the optimum pH condition and for the photo-Fenton process in a pH range 3.0 to 6.0.

Process	pH	*k*_app_ (min^−1^)	Residual TOC Concentration
Photocatalysis	6.0	9.0 × 10^−3^	81%
Fenton	4.0	3.2 × 10^−2^	65%
Photo-Fenton	3.0	1.5 × 10^−1^	21%
Photo-Fenton	4.0	1.3 × 10^−1^	30%
Photo-Fenton	5.0	1.2 × 10^−1^	30%
Photo-Fenton	6.0	6.2 × 10^−2^	63%

### 2.6. Stability and Reusability of BFO in the Photo-Fenton System

The stability and reusability of BFO in the photo-Fenton system was evaluated in four consecutive runs at pH = 4.0. The catalyst was not dried or washed between the cycles in order to adapt to conditions realistic for the application. Five quartz tubes under the same test conditions above were sampled by pouring all of the solution into a centrifuge tube at 0, 10, 30, 60 and 120 min, respectively. The solution was centrifuged to separate BFO and the supernatant. BFO was reused, and the supernatant was analyzed by chromatography, TOC and ICP-OES measurement.

**Figure 7:**
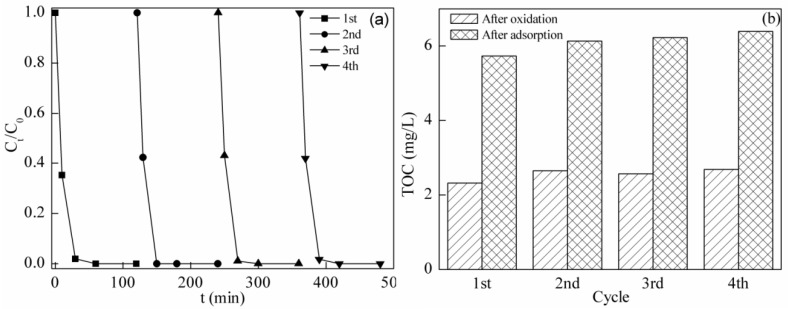
(**a**) Degradation of TC in successive cycles by the photo-Fenton process; (**b**) Residual TOC concentration after adsorption and the photo-Fenton process of each cycle.

TC was degraded within 60 min, and the final TOC concentration was stable, as is shown in Figure 7a,b. No leaching of Fe and Bi ions was detected. The results reveal that BFO appears to be stable and reusable, which is in accordance with the reported result [20]. The classic homogeneous Fenton system that uses iron ions as a catalyst will produce large amounts of iron sludge. The heterogeneous BFO photo-Fenton system will not generate iron ions. It is promising to overcome the problem of sludge production by replacing the classic Fenton system with the photo-Fenton system.

### 2.7. Removal of TC Using Different Processes

Advanced oxidation processes (AOPs) using different systems and materials have been tested to remove TC in water. A comparison was summarized among different AOPs removing tetracycline in recent literature in Table 2. As is shown, the BFO photo-Fenton system shows a good performance in this study. The reaction rate and TOC removal of the BFO photo-Fenton process are quite excellent. AOPs involving the Fenton process reveal better performance than those with photocatalysis alone, according to the results of the rate constants. The results also show that the photo-Fenton process has a much better performance than the photocatalytic or Fenton-like processes. The combination of different kinds of AOPs into one system may greatly boost the oxidation effect. The BFO photo-Fenton system is promising to be put into application.

**Table 2:** Comparison of different advanced oxidation processes (AOPs) for removing TC.

Initial TC Concentration (mg/L)	Degradation Systems	Optimal Apparent Rate Constant (min^−1^)	Optimal Residual TOC after 120 min	Type of Catalyst	Reference
55.0	Photocatalysis	6.6 × 10^−2^	NG *	TiO_2_	[1]
10.0	Photocatalysis	1.6 × 10^−2^	NG	SrTiO_3_	[48]
20.0	Photocatalysis	3.4 × 10^−2^	NG	Sr-Bi_2_O_3_	[12]
20.0	Photocatalysis	9.6 × 10^−2^	NG	MWNTs–Bi_2_WO_6_	[49]
10.0	Photocatalysis	9.9 × 10^−3^	NG	AgIn(MoO_4_)_2_-Ag/Ag	[50]
10.0	Photocatalysis	1.7 × 10^−2^	NG	SrTiO_3_	[51]
10.0	Photocatalysis	4.4 × 10^−3^	85%	Ni_(1−*x*)_Cu_(*x*)_Fe_2_O_4_	[52]
0.02	Photo-electro-Fenton	2.8 × 10^−2^	70%	Fe_3_O_4_-graphite	[53]
100.0	Electro-Fenton	1.8 × 10^−1^	15%	Boron-doped diamond(BDD)/carbon-felt electrode, Fe^3+^, Fe^2+^	[54]
100.0	Electro-Fenton	2.7 × 10^−1^	58%	carbon-felt electrode, Fe^2+^	[55]
10.0	Photo-Fenton	1.5 × 10^−1^	21%	BFO	this article

* NG, not given.

## 3. Experimental Section

### 3.1. Materials and Reagents

Bismuth nitrate (Bi(NO_3_)_3_·5H_2_O), iron nitrate (Fe(NO_3_)_3_·9H_2_O), potassium hydroxide (KOH) and potassium nitrate (KNO_3_) from Alfa Aesar were used without further purification. Nitric acid (HNO_3_), 30% hydrogen peroxide (H_2_O_2_) solution, ethanol and tetracycline (TC) were bought from Sinopharm. Ultrapure water used in the experiment was produced from a Milli-Q ultrapure water system.

### 3.2. Preparation of BFO

BFO was synthesized by the alkaline hydrothermal method. One-point-five grams of Bi(NO_3_)_3_·5H_2_O and 1.2 g of Fe(NO_3_)_3_·9H_2_O were added to 5.0 mL of 10% HNO_3_ solution. The pH of the solution was adjusted to 10.0 by adding 12.0 M KOH solution dropwise under magnetic stirring. The resulting coprecipitate of Fe(OH)_3_ and Bi(OH)_3_ was washed several times with ultrapure water by centrifugation until the pH value of the supernatant reached 7.0. Then, 36.0 mL of 4.0 M KOH solution were added after the supernatant was discharged, and this mixture was treated under ultrasonication for some time to let the dense coprecipitate disperse in the solution homogeneously. The resulting suspension was transferred into a 50-mL Teflon-lined stainless steel autoclave into which 6.1 g KNO_3_ had been previously added. The mixture was magnetically stirred for 30 min and then heated at 160 °C for 12 h. Then, BFO material was gathered and washed several times with ultrapure water and ethanol. Finally, the resulting material was dried at 60 °C and ground into powder. Overall, this is a simple method with mild reaction conditions.

### 3.3. Analytical Methods

To detect the concentration of tetracycline, ultrahigh performance liquid chromatography (UPLC) (Waters, H-class, Singapore) equipped with a Tunable ultra violet (TUV) detector at 278 nm and a Waters BEH column (C18-1.7 µm, 2.1 × 50 mm) were used. A mobile phase containing water (0.1% formic acid) and acetonitrile with a volume ratio of 80/20 was maintained for 4 min with a flow rate of 0.2 mL·min^−1^ at a 313 K column temperature. Total organic carbon (TOC) was measured by a TOC analyzer (Shimadzu, TOC-L cph). The X-ray powder diffraction (XRD) pattern of BFO was obtained with a diffractionmeter (Bruker, D8 Advance, Karlsruhe, Germany). Scanning electronic microscope (SEM) images were from a Hitachi S-3400 SEM. The UV-VIS spectrum was obtained from a UV-VIS spectrophotometer (Shimadzu, UV-2550, Suzhou, China). The zeta potential and size distribution of BFO were measured by a Zetasizer (Malvern, ZS90, Malvern, UK). The concentrations of Fe and Bi elements were measured by ICP-OES (Agilent, 720ES, Palo Alto, CA, USA).

### 3.4. Degradation Experiments

A 500-W xenon lamp is located in the center of the reactor within a double-walled cooling quartz well of 5 cm in diameter. Several quartz tubes hold the reaction solution. The light path is 80 mm. The visible light photocatalysis, Fenton and photo-Fenton processes for removing TC were investigated with BFO. As for the photocatalytic degradation, the activity of BFO was evaluated under visible light irradiation with UV cut-off filters to remove any irradiation below 420 nm. The residual TC concentration by UPLC measurement was referred to as *C*_t_/*C*_0_, where *C*_t_ is the TC concentration at *t* = *t* and *C*_0_ is that at *t* = 0.

Concentrations of BFO and initial pH values were adopted as variables in the photocatalytic experiment. Prior to the photocatalytic reaction, the suspension solution was magnetically stirred in the dark for 60 min to reach the adsorption equilibrium. Then, the light was turned on, and samples were taken at selected time intervals with 20.0 μL of isopropanol added to quench the radicals inside the system. Photocatalysts were removed by filtration with 0.22-μm syringe filters, and the supernatant was gathered for the UPLC and TOC detection.

For the Fenton experiment, the best dosage of BFO, H_2_O_2_ concentration and initial pH values were optimized. Prior to the Fenton reaction, the solution was magnetically stirred in the dark for 60 min to reach the adsorption equilibrium. Then, H_2_O_2_ solution was added in, and samples of 0.5 mL were taken at selected time intervals with another 20.0 μL of isopropanol and 0.5 mL of 1.0 M Na_2_S_2_O_3_ solution added to quench the radicals and residual H_2_O_2_ inside the reaction system. Finally, the photocatalysts were removed by filtration with 0.22-μm syringe filters, and the supernatant was gathered for the UPLC and TOC detection. The photo-Fenton experiment was tested using concentration parameters predetermined to test the performance of a process combining photocatalytic and Fenton degradation together. The influence of initial pH values on the reaction system was studied. The sampling and measurement of the degradation performance of the photo-Fenton process were the same as the Fenton experiment.

## 4. Conclusions

BFO has been successfully synthesized as a photocatalyst and a heterogeneous Fenton catalyst by a facile and mild hydrothermal method in this study. The BFO dosage was determined by a photocatalysis test, and the H_2_O_2_ dosage was determined by a Fenton-like test. The oxidation effect of both the photocatalysis and Fenton-like systems was affected by pH. The mechanism of change in the oxidation effect with the variation of different parameters was discussed. UPLC was used together with TOC measurement to evaluate the degradation efficiency of TC. The TOC measurement got rid of the influence from photolysis and enriched the evaluation method. The BFO photo-Fenton system largely improved the oxidation efficiency compared to that achieved by the photocatalysis or Fenton systems. It also extended the operating pH range to a higher value. The photo-Fenton system appears to achieve higher efficiency at higher pH than the classical Fenton system. There was no leakage of metal ions, which make it promising in overcoming the problem of sludge production after oxidation. Our findings showed that the BFO-catalyzed photo-Fenton system had good potential in antibiotic-containing waste water treatment.
